# Apple-marigold intercropping improves soil properties by changing soil metabolomics and bacterial community structures

**DOI:** 10.3389/fmicb.2023.1195985

**Published:** 2023-06-29

**Authors:** Xiaomin Xue, Ru Chen, Chao Xu, Chunxiang Zhang, Lijuan Dong, Xianyan Zhao, Xiaohan Wang

**Affiliations:** ^1^Shandong Institute of Pomology, Tai’an, China; ^2^School of Bioengineering, Qilu University of Technology, Shandong Academy of Sciences, Jinan, China; ^3^Taishan Forestry Research Institute, Tai’an, China

**Keywords:** marigold, intercropping, soil properties, soil metabolomics, bacterial community structures

## Abstract

Marigold can protect crops against soil-borne diseases. However, the effects of intercropping with marigold on apple rhizosphere soils are not known. In this study, we investigated the metabolite profiles and bacterial community structures in rhizosphere soils of the apple-marigold intercropping system by high-throughput sequencing and soil metabolomics. The results show that intercropping marigold could significantly enhance soil moisture, nitrogen, and enzyme activities compared with clean tillage. The soil metabolite profiles and the soil bacterial community structures in the rhizosphere soils were different between the inter-and mono-cropping systems. Among nine metabolites, carbohydrates were more increased in the intercropping system than in the monocropping system. Pathway enrichment analysis revealed that the greatest differential, in terms of metabolic pathway, was starch and sucrose metabolism. Moreover, intercropping marigold significantly increased the relative abundance of plant growth promoting bacteria in rhizosphere soils, such as Rhizobiales, Pseudomonadales, and Bacillales. These results indicate that marigold intercropping positively affected the apple orchard’s soil quality and may provide a new intercropping technique to improve soil fertility in orchards and promote plant growth.

## Introduction

The apple (*Malus pumila* M.), rich in nutrients such as dietary fiber, vitamins and trace elements, is one of the most nutritious and highest-value fruits. The apple industry plays a critical role in the world economics, and its planting area and annual output reached 4.62 million ha and 86.4 million tons by 2020 (FAOSTAT), respectively. In particular, China’s apple planting area and output accounted for >50% of the global total in 2017 (FAOSTAT). However, long-term planting and excessive use of inorganic fertilizers and pesticides may lead to a decline in apple quantity and quality, negatively impact soil quality, and cause leaching and pollution of groundwater (Wang et al., 2016). Therefore, it is necessary to develop green, practical, and cost-effective methods for managing apple orchards.

Intercropping, which involves growing two or more crops in the same field, is a widely used agricultural practice (Wang et al., 2015; Raza et al., 2021). It can enhance soil moisture, enzyme activities and soil fertility, and reduce the impact of soil-borne diseases and pests, thus improving soil quality and improving the yield and quality of crops (Weerarathne et al., 2017; Raza et al., 2020; Li et al., 2021). Apple-based intercropping has been commonly applied owing to its numerous benefits for soil and crops and its role in the promotion of sustainable agricultural development (Gao et al., 2022; Zheng et al., 2022). Compared to monoculture, the apple-white clover intercropped system had larger apple root systems and white clover biomass (Yang et al., 2019). In addition, apple-ryegrass intercropping enhanced the total soil organic carbon and dissolved organic carbon content and improved soil fertility and quality (Zhang et al., 2019). Marigold (*Tagetes erecta* L.), a member of the Asteraceae family, is an aromatic medicinal plant and is often intercropped due to its bactericidal, fungicidal, insecticidal, and herbicidal qualities (Asrar and Elhindi, 2011; Santos et al., 2015). Intercropping with marigold has been found to increase soybean yield (El-Hamawi et al., 2004). In another study, the rhizosphere fungal community structure of angelica was significantly affected when intercropped with marigold (Wei et al., 2015). However, few studies have focused on the apple-marigold intercropping system.

The rhizosphere harbors a large number of microbes, and is the most important interface for soil–plant-microbe interaction. Plant roots release exudates, which are the main nutrient source for bacteria, driving their communities and activities (Pii et al., 2016). Soil microorganisms have a significant effect on soil properties, nutrient cycle, plant growth and crop quality (Sahu et al., 2017). Previous studies have demonstrated the changes in the microbial characteristics of soil microbial caused by intercropping, such as increasing the abundance of *Rhizobium hainanense* and *Rhizobium leguminosarum,* which were involved in nitrogen fixation in the maize-peanut intercropping system (Chen et al., 2018). In addition, the diversity of soil bacterial communities was increased by means of trifolium-cucumber and mustard-cucumber intercropping systems (Li and Wu, 2018). However, no study has yet been conducted on the effects of intercropping marigold on the bacterial structure, composition and diversity of apple orchard soil rhizosphere.

Most studies of intercropping have focused on changes in microbial communities and their interactions with soil properties, while soil metabolites are rarely discussed. Soil metabolites, exuded and transformed by plants and soil microbes, play an important role in regulating plant growth (Canarini et al., 2019). Soil metabolomics, a tool which is used increasingly in the related literature, can be highly informative with regard to soil biology, plant root secretions and the behavior of soil bacteria (Zhao et al., 2019). Analysis of soil metabolites can elucidate soil chemistry, which may be exploited to improve soil and apple quality. For example, *Ocimum basilicum* L. and *Satureja hortensis* L. intercropping can change the microbial structures and functional groups connected with the N cycle during soil organic matter (SOM) mineralization of the rhizophere soil of pear trees by the main root exudates, such as saccharides and amine, produced by these aromatic plants (Zhang et al., 2021). However, changes in soil metabolites and bacterial composition in an apple-marigold intercropping system, are less well understood.

Thus, in this study, we investigated the effects of intercropping marigold on soil bacterial structures and soil metabolomics in an apple orchard and established the relationships between the rhizosphere chemistry and the bacterial community through soil metabolomics. These data are essential for evaluating the effectiveness of marigold as an intercrop in improving the soil quality of apple orchards.

## Materials and methods

### Overview of the experimental orchard

The experiment was conducted from 2021 to 2022. The experimental orchard was located at the Tianping Lake Base of Shandong Institute of Pomology (N latitude: 36°12′55.36″, E longitude: 117°01′09.87″) at an altitude of 168 m. The sunshine duration of the region is 2627.1 h, the annual average precipitation is 697 mm, the annual average temperature is 12.9°C, and the annual cumulative temperature (≥0°C) is 47,319°C. The average frost-free period is 195 d. Modern dwarf anvil intensive cultivation was used, with “Tian Hong 2/SH38/eight-angled begonia” as a test tree. The orchard was planted in the spring of 2010, with a spacing of 0.75 m × 4.0 m. The soil was sandy loam with medium fertility. The orchard irrigation conditions were well established. The management level of the orchard was above average. The orchard was well-organized, and the apple trees were healthy with normal growth.

### Experimental design

The experimental treatment began in the spring of 2021. A total of two treatments were set up in the experiment: (i) an intercropping system (T), *Tagetes erecta* L. was planted between rows of apple trees and (ii) a control, clean tillage (CK) between rows. Single rows plot design was adopted and repeated 3 times, approximately 0.2 hm^2^. The grass seeds for the test were provided by the aromatic plant research group of Beijing Agricultural University. They were sown in the ditch in April. The depth of the ditch was about 2.0 cm, and the distance between the ditches was 20 cm. Five rows were planted between each row. Weeds were removed early in seedling emergence to keep the grass growing area free of weeds. When the grass height is above 40 cm, it should be mowed, cut twice in 2021 and four times in 2022. The field management measures for each treatment are carried out according to conventional methods.

### Soil physicochemical properties and enzyme activity analysis

Soil samples were collected in late October 2022. A soil sampler was used to collect soil at a depth of 15 cm within a 25-cm radius of the tree root. The “S”-shaped method was adopted to randomly select 6 apple trees with basically the same growth. According to the five-point sampling method, soil sampling was performed at five points and mixed as one composite sample. Three replicates were used to minimize spatial heterogeneity-related inaccuracy (Farooq et al., 2022). Root systems were extracted from land and the outer layer soil was shaken off from roots. Soil still attached to root surface were gently gathered with a sterilized brush as rhizosphere soil. Soil samples were placed in sealed bags, put on dry ice, and returned the laboratory. Each sample was divided into two subsamples. One subsample was air-dried and used to determine soil physicochemical properties and enzyme activities. The other subsample was stored at −80°C for microbial community and metabolism analyses.

Soil pH, organic matter (OM), total nitrogen (TN) content, total phosphorus (TP), available nitrogen (AN) and available phosphorus (AP) were determined according to the method outlined using Bao (2000). Soil moisture content was measured by the drying method (Zhang et al., 2018). Soil sucrase, urease, phosphatase, and cellulase activities were determined by 3,5-dinitrosalicylic acid, indophenol blue, benzodisodium phosphate colorimetry and anthrone, respectively.

### High-throughput sequencing

The DNA from different samples was extracted using a FastDNA SPIN Kit (MP Biomedicals, Santa Ana, CA, United States). The V5-V7 region of the bacterial 16S rRNA gene was amplified using primers 799F (5′-AACMGGATTAGATACCCKG-3′) and 1193R (5′-ACGTCATCCCCACCTTCC-3′) (He et al., 2022; Sun et al., 2022) following the protocol: each 25 μL system containing 12.5 μL PCR premix (Ex TaqTM; Takara, Shiga, Japan), 0.5 μL (10 μM) of the forward and reverse primers (final concentration of each primer 0.2 pmol μl^−1^), 0.5 μL DNA template (20 ng), and 11 μL sterile double distilled water. The following thermocycling conditions were used: initial denaturation at 94°C for 10 min, 30 cycles of denaturation at 94°C for 30 s, annealing at 55°C for 45 s, and extension at 72°C for 1 min, followed by a final extension at 72°C for 10 min. High-throughput sequencing was performed using the Illumina HiSeq2500 platform (Illumina, San Diego, CA, United States). The sequencing data obtained in this study have been deposited in the National Center for Biotechnology Information (accession No. PRJNA916681).

Bioinformatics analyses were performed using VSEARCH (version 2.21.1) according to previously described methods (Sun et al., 2022). Low-quality sequences (Phred quality score < 20, length < 200 bp) and chimeras were filtered out. The clean sequences were subsequently denoised using the UNOISE algorithm (version 3) and generated zOTUs (zero-radius operational taxonomic units). Taxonomic assignment was performed using SINTAX based on SILVA 16rRNA database (Sun et al., 2023) Bacterial α diversities were calculated from the representative OTU table in QIIME (v1.9.1) (Caporaso, 2010). Principal coordinate analysis (PCoA) was performed based on Bray-Curtis distance using R v.3.2.1 with the vegan package. Soil bacterial taxa (genera) of apples that differed significantly in terms of relative abundance in mono- and intercropped systems were identified as potential biomarkers by the linear discriminant analysis (LDA) effect size (LEfSe) analysis.^1^ Taxa were identified at genus level using the following parameters: (1) α-value = 0.05 for factorial Kruskal-Wallis tests among classes and (2) threshold logarithmic LDA score > 2.0 for differential features (Bourhane et al., 2023).

### Soil metabolite analysis

The soil metabolite extraction method was modified from Duan et al. (2022) Briefly, approximately 0.5 g soil sample was mixed with 0.5 mL of methanol: isopropanol: water (3,3:2 V/V/V) mixture, vortexed for 3 min and subjected to ultrasonication for 30 min. The extract was then centrifuged at 12000 rpm under 4°C for 3 min and the supernatant was mixed with internal standard and evaporated under a nitrogen gas stream. The sample was freeze-dried in a lyophilizer, and the residue was used for further derivatization. The residue was mixed with 0.1 mL of methoxyamine hydrochloride in pyridine (15 mg·mL^−1^) and incubated at 37°C for 2 h. Then 0.1 mL of Bis(trimethylsilyl)trifluoroacetamide (with 1% Trimethylchlorosilane) was added into the mixture and kept at 37°C for 30 min after vortex-mixing. The resulting solution (1 μL) was detected by a gas chromatograph (Agilent 8890) coupled to a 5977B mass spectrometer with a DB-5MS column (30 m length × 0.25 mm i.d. × 0.25 μm film thickness, J&W Scientific, United States) (GC–MS). Helium, as a carrier gas, was accessed at a constant flow rate of 1 mL·min^−1^. The initial temperature was kept at 40°C for 1 min, then raised to 100°C at 20°C min^−1^, then raised to 300°C at 10°C min^−1^, and then kept at 300°C for 5 min.

The original data obtained by GC–MS analysis was first extracted by MassHunter software (Agilent) to obtain the mass-to-charge ratio, retention time, peak area and other information of the characteristic peak, and then the data was statistically analyzed. A series of preparations and organization on the original data were conducted: (1) retention index calculation, (2) filter a single peak and only retain peak area data with no more than 50% empty values for a single group or no more than 50% empty values for all groups, and (3) data normalization, using internal standard normalization. Significantly regulated metabolites between groups were determined by VIP ≥ 1 and absolute Log2FC (fold change) ≥ 1. VIP (Variable important in projection) values were extracted from the orthogonal partial least squares discriminant analysis (OPLS-DA) results, containing score plots and permutation plots generated using R package MetaboAnalystR. The data were log-transformed (log2) and mean-centered before OPLS-DA. A permutation test (200 permutations) was performed to avoid overfitting.

### Statistical analyses

Kruskal-Wallis tests (*p* < 0.05) were used to compare the treatment means. Statistical analyses were performed using the SPSS 20.0 software (SPSS Inc., United States).

## Results

### Soil properties and enzyme activities

Compared to the control, intercropping marigold increased the contents of soil available alkali-hydrolysis nitrogen (AN) and potassium (AP), organic matter (OM), and moisture by 35, 24, 25, and 53%, respectively (Table 1). The activities of soil urease, phosphatase, sucrase, and cellulase were also higher in the treatment with intercropping marigold (Table 1). However, there were no significant differences in the soil pH and total contents of nutrients (TN and TP) between the inter-- and the mono-cropping systems (Table 1).

**Table 1:** Effects of intercropping marigold on soil physico-chemical properties of apple orchard.

	Clean tillage	Intercropping
pH	6.77 ± 0.05	6.91 ± 0.05
OM (g kg^−1^)	16.41 ± 0.42	20.47 ± 0.10*
TN (g kg^−1^)	0.73 ± 0.03	0.80 ± 0.01
TP (g kg^−1^)	0.71 ± 0.11	0.73 ± 0.02
AN (mg kg^−1^)	40.00 ± 3.23	53.97 ± 3.69*
AP (mg kg^−1^)	4.48 ± 0.29	5.54 ± 0.48*
Soil moisture content (%)	13.17 ± 1.56	20.09 ± 0.47*
Urease (mg g^−1^ d^−1^)	0.75 ± 0.03	0.85 ± 0.03*
Phosphatase (mg g^−1^ d^−1^)	0.82 ± 0.04	1.20 ± 0.02*
Sucrase (mg g^−1^ d^−1^)	15.81 ± 0.57	26.42 ± 0.21*
Cellulase (mg g^−1^ d^−1^)	5.17 ± 0.09	7.72 ± 0.23*

OM, organic matter; TN, total nitrogen; TP, total phosphorus; AN, alkali-hydrolysis nitrogen; AP, available phosphorus. Data (means ± SD, *n* = 3) followed by asterisks indicated significant differences between treatments at *p* < 0.05 verified by Kruskal-Wallis tests.

### Soil bacterial community

**Figure 1:**
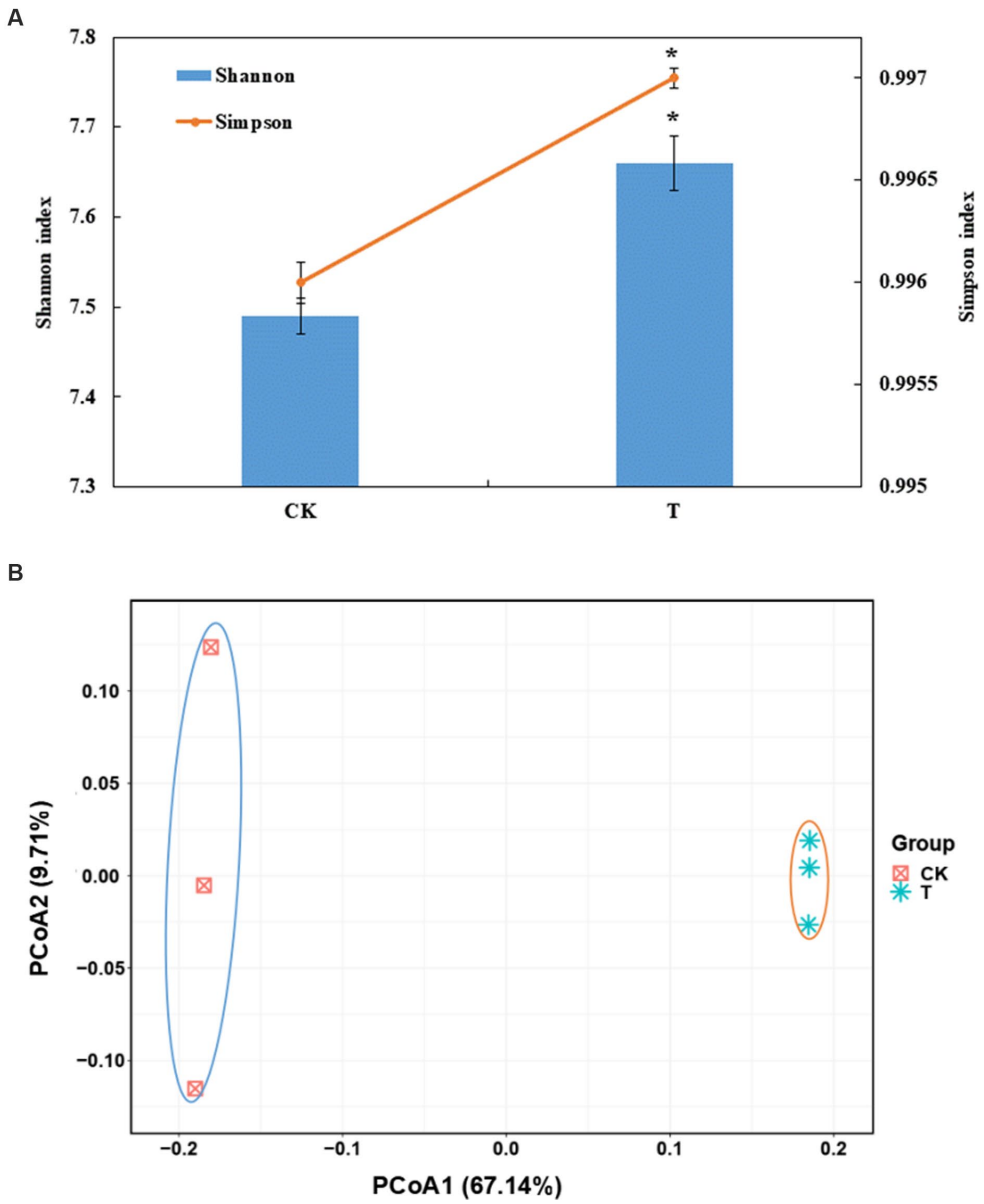
**(A)** Effects of intercropping marigold on the α-diversity indices of the bacterial communities in the rhizosphere soils of apple orchard. Data are means of three replicates. Error bars represent standard deviation. Asterisks indicate significant differences between treatments at *p* < 0.05 verified by Kruskal-Wallis tests. **(B)** Microbial community composition by PCoA (principal co-ordinates analysis) at the OTU level, based on 97% sequence similarity. T, intercropping system; CK, monocropping system.

**Figure 2:**
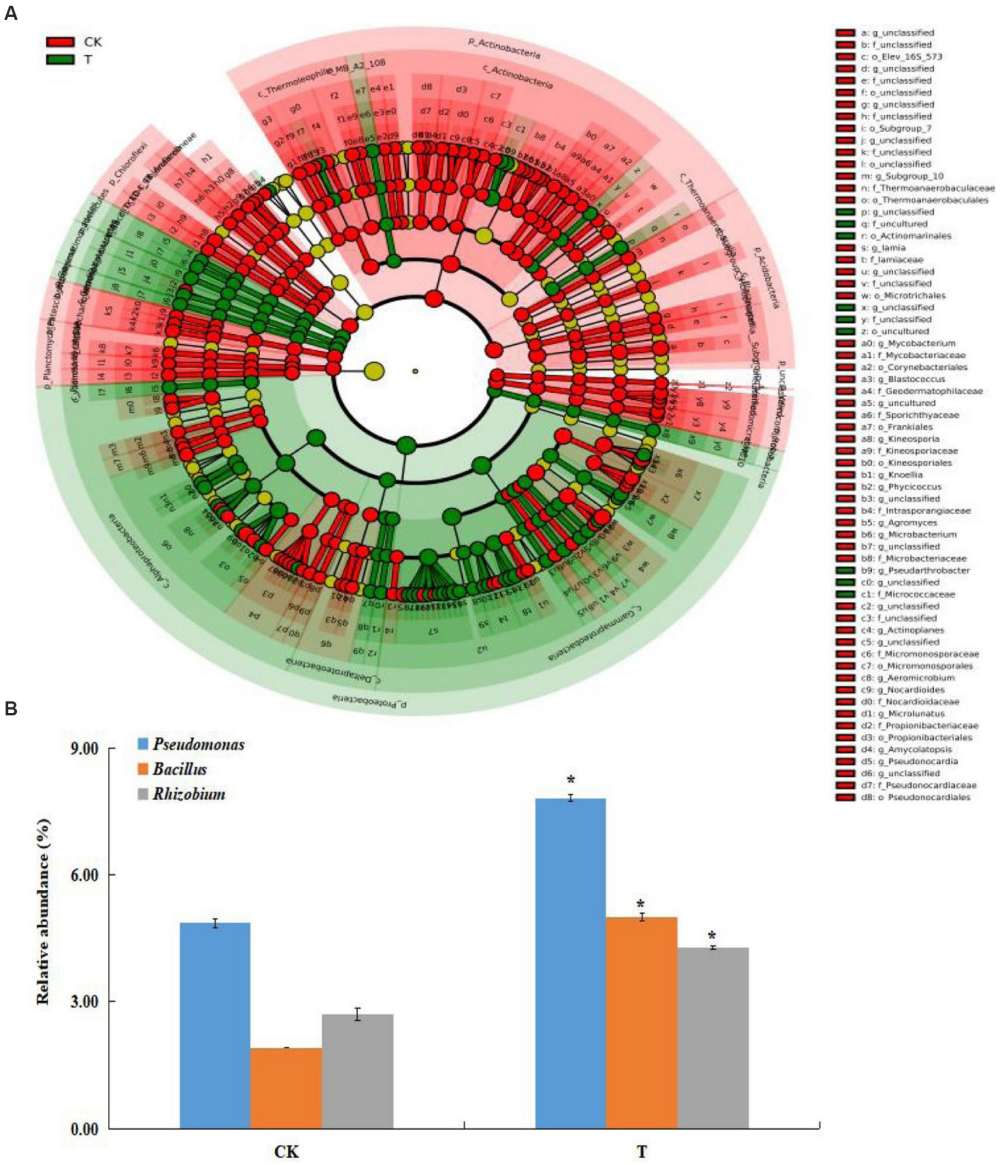
**(A)** Cladogram indicating the polygenetic distribution of bacterial lineages at genus level in the intercropping systems as determined by LefSe analysis (LDA effect size analysis). Each circle’s diameter is proportional to the taxon’s abundance. **(B)** The effects of intercropping marigold on the relative abundance of genera of the apple orchard soil. Data are means of three replicates. Error bars represent standard deviation. Asterisks indicate significant differences between treatments at *p* < 0.05 verified by Kruskal-Wallis tests. T, intercropping system; CK, monocropping system.

A total of 608,722 OTUs, with a mean of 101453OTUs per sample (min = 69675 and max = 130662), were obtained from the soil samples (Supplementary Table S1). The Shannon and Simpson indexes were significantly increased in the marigold intercropping system compared with the control (Figure 1A). According to principal coordinate analysis (PCoA), significant differences between the intercropping and monocropping systems were associated with the first coordinate axis. The first two axes presented a total difference of 76.85% (Figure 1B). Abundant groups with relative abundances >1% were constitutive of 11 phyla. Proteobacteria was the dominant phyla (43.5 and 52.8% in CK and T), followed by Actinobacteria (27.5 and 19.4%, respectively) (Supplementary Figure S1A). At the order level, Betaproteobacteriales, Rhizobiales, Micrococcales, Pseudomonadales and Bacillales were dominant. Among these, the relative abundance of Rhizobiales, Pseudomonadales and Bacillales were significantly increased by intercropping marigold compared to the control (Supplementary Figure S1B). Similar results were also observed in genera Pseudomonas, Bacillus, and Rhizobium (Figure 2B). LEfSe analysis showed that the Proteobacteria and Actinobacteria were the marker bacterial phyla in intercropping and monocropping systems, respectively (Figure 2A). In addition, Pseudomonas, Bacillus, Nitrospira and Bradyrhizobium were the marker bacterial genera in the intercropping system (Figure 2A).

### Soil metabolites

**Figure 3:**
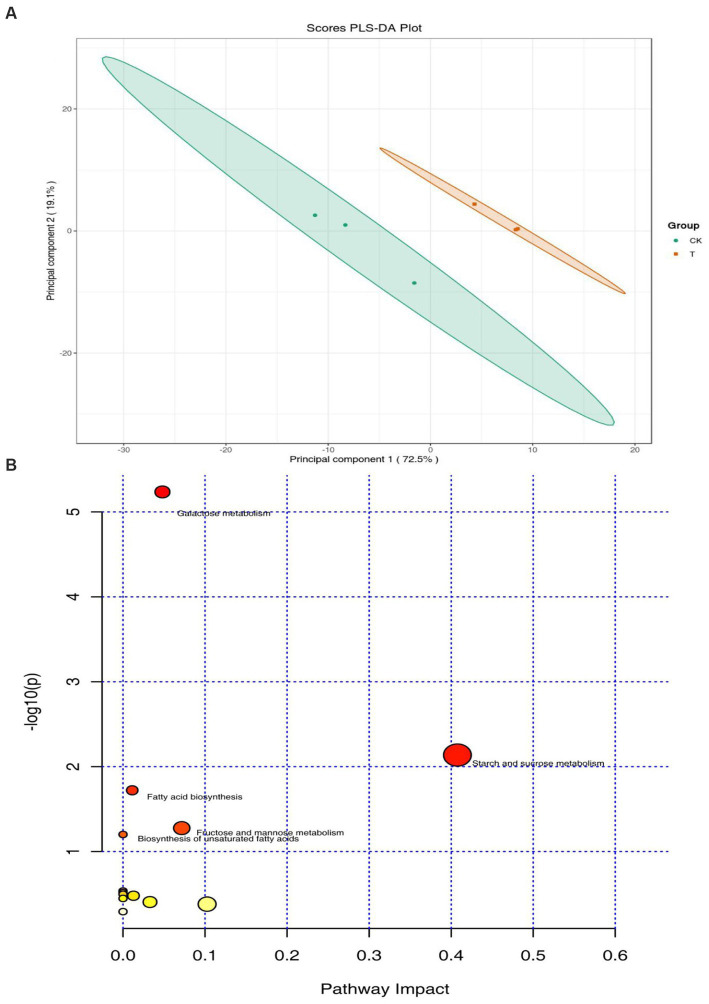
Distribution of soil metabolites in the intercropping system (T) and monocropping system (CK). **(A)** Principal component analysis (PCA) score plots of soil metabolome. **(B)** A bobble diagram of metabolic pathways based on the different metabolites.

A total of 133 peaks were detected in the chromatogram. Based on the agreement of the mass spectrum fingerprint and the retention index, 80 metabolites in the rhizosphere soils were identified and grouped into 4 classes, namely organic acids, carbohydrates, lipids, and others (phenol, amine). Of these compounds, the concentration of lipids was the greatest, accounting for 45% of the total, followed by carbohydrates (18%). Partial least-squares discriminant analysis (PLS-DA) showed a clear separation between the intercropping system and the monocropping system. The first and second principal components accounted for 72.5 and 19.1% of the total variation, respectively (Figure 3A). The heatmap demonstrated that lipids and carbohydrates were enriched in the intercropping system (Supplementary Figure S2). Furthermore, 17 metabolites showed significant differences between the intercropping and monocropping systems (Table 2). Among these, 9 metabolites were upregulated and 8 were downregulated (Table 2). Of the 8 differential carbohydrates, 6 showed higher expression in the intercropping system than in the monocropping system (Table 2). Pathway enrichment analysis on these differential metabolites was used to illuminate the specific changes in soil metabolic pathways. Starch and sucrose metabolism was the most significantly altered process in rhizosphere soil (Figure 3B). Fructose and mannose metabolism and galactose metabolism were also significantly impacted (Figure 3B).

**Table 2:** Soil metabolites that were significantly different between the intercropping system and monocropping system.

Compounds	Class	*p*-value	Type
1-(diisopropylphosphino)-3-(diisopropylphosphinyl)-Propane	Others	3.60 × 10^−3^	Upregulated
Propanedioic acid	Lipid	1.91 × 10^−2^	Upregulated
2,4-Di-tert-butylphenoxytrimethylsilane	Other	1.13 × 10^−3^	Upregulated
D-Threitol, 4TMS derivative	Carbohydrate	9.06 × 10^−3^	Downregulated
D-(−)-Ribofuranose	Carbohydrate	6.66 × 10^−3^	Upregulated
D-Fructofuranoside	Carbohydrate	3.22 × 10^−2^	Downregulated
Propanamide	Other	2.82 × 10^−2^	Downregulated
Myristic acid, TMS derivative	Organic acids	1.58 × 10^−4^	Downregulated
D-Fructose	Carbohydrate	8.99 × 10^−2^	Upregulated
D-Glucose	Carbohydrate	6.25 × 10^−2^	Upregulated
D-(+)-Talose	Carbohydrate	6.24 × 10^−3^	Upregulated
D-Allose	Carbohydrate	7.31 × 10^−2^	Upregulated
D-Glucitol, 6TMS derivative	Carbohydrate	7.78 × 10^−2^	Upregulated
9(E),11(E)-Conjugated linoleic acid, tert.-butyldimethylsilyl ester	Lipid	1.72 × 10^−2^	Downregulated
Stearic acid, TMS derivative	Lipid	3.32 × 10^−2^	Downregulated
(Z)-Docos-13-enamide, N-TMS	Other	3.72 × 10^−3^	Down

## Discussion

The facilitative interactions in intercropping systems that can improve the nutrient and water acquisition of soil are complicated. It is important to elucidate these interactions for the practical application of any sustainable agroecosystem (Homulle et al., 2022). To our knowledge, the apple-marigold intercropping system and its influence on apple orchard soil quality have not been studied. The present work studied the properties, metabolites and bacterial community of soils in the apple-marigold intercropping system to clarify the effects of marigold on apple rhizosphere soils. It was demonstrated that intercropping marigold could significantly increase and maintain the moisture content of apple rhizosphere soils by providing soil shading (Table 1). Mulching practice plays an important role in decreasing soil evaporation (Zhang et al., 2022). Soil nutrients are closely related to fruit yield and quality and sustainable production in orchards (Wei et al., 2017). Intercropping marigold could significantly improve the soil organic matter, available phosphorus, and available nitrogen contents (Table 1). This indicates that marigold roots may mediate the soil nutrients and influence nutrient cycling in the soil. Li et al. (2020) found an evident increase in soil nutrients such as organic matter and available phosphorus in tobacco-marigold intercropping system. Soil enzyme activities can influence soil nutrient cycling and soil property and fertility level (Cheng et al., 2020). Soil enzymes are affected by soil moisture, microbes, nutrients, and other factors. In this study, apple-marigold intercropping resulted in significant increases in the urease, phosphatase, sucrase and cellulase activities of rhizosphere soils (Table 1). These findings are consistent with the results of intercropping plants to improve the enzyme activity in rhizosphere soils of apple, tea and kiwifruit (Qian et al., 2014; Ma et al., 2017; Wang et al., 2021). Generally, the improvement of soil moisture, nutrients and enzyme activities by intercropping marigold in this study resulted in an improved environment for the growth of apples.

The community structure and diversity of soil bacteria are important biochemical indicators of soil health, which can be affected by agriculture management (Li et al., 2022; Zhao et al., 2022). Intercropping can improve the quantity of orchard soil microorganisms which promote plant growth, enhance plant defenses, and inhibit soil-borne diseases (Wang et al., 2020; Yuan et al., 2023). In this study, intercropping marigold improved soil moisture, nutrients and enzyme activities, which may affect rhizosphere organisms, and then influence the structure and diversity of the soil bacterial community (Cui et al., 2018). We found that the bacterial Shannon and Simpson indexes were higher in the apple-marigold intercropping system compared with monocropping system (Figure 1A), suggesting that apple-marigold intercropping improved soil bacterial diversity and richness. Microbial communities with a high alpha diversity index are generally more complex and stable (Yang et al., 2022). Moreover, there was a clear separation between different cropping systems (Figure 1B), indicating a significant shift in the apple rhizosphere bacterial community composition when intercropped with marigold. Similarly, Li et al. (2020) found that marigold intercropping improved the alpha and beta diversity of tobacco rhizosphere soil bacteria compared to a monocropping system. Among the marker bacteria associated with marigold intercropping, Proteobacteria and Actinobacteria can enhance nutrient availability and plant growth regulators (Kour et al., 2019). The orders Rhizobiales and Pseudomonadales, which belong to the Proteobacteria phylum, were improved in apple-marigold intercropping system rhizosphere soils, as well as the order Bacillales (Figure 2A). These orders are mainly enriched in the rhizosphere and decompose organic matter to promote soil carbon and nitrogen cycles (Zhou J. et al., 2020; Pang et al., 2021). Thus, their enrichment may improve soil nutrients and promote plant growth.

Soil metabolites were derived from plant root exudates, microbial metabolites, as well as from plants, microbes and soil organic decomposition (Ni et al., 2021). Differential metabolites, sensitive to the marigold intercropping, were first investigated. The main differential compounds were carbohydrates in this research (Table 2). In the rhizosphere, carbohydrates are the main drivers of shifts in bacterial communities, constitute the majority of organic matter, and contribute greatly to carbon stock and aggregate stability (Hazarika et al., 2009; Guidi et al., 2015; Zhou S. et al., 2020). They are, therefore, used as an indicator of soil physical quality. In this study, 75% differential of carbohydrates, including D-Fructose and D-Glucose, were upregulated, suggesting that marigold intercropping may improve soil quality by regulation of soil metabolome. The specific changes in soil metabolic processes were elucidated by pathway enrichment analysis. Starch and sucrose metabolism, fructose and mannose metabolism, and galactose metabolism were significantly altered pathways in rhizosphere soil (Figure 3B). Fructose and glucose have been found to enhance innate immunity against bacterial and microbial pathogens in plants (Peinado et al., 2013; Qian et al., 2015). Overall, marigold intercropping provided more carbohydrates for plant growth promotion than the control. However, the precise contribution of carbohydrate enrichment to plant growth requires further investigation. There is a significant relationship between soil metabolites and soil microbial communities which can guide soil fertility conditions. The increase in carbohydrates may have reduced the abundance of Chloroflexi and Actinobacteria, which are potential opportunities to against microbial pathogens (Huang et al., 2020; Lu et al., 2022). Further research is required to determine how soil metabolites affect soil ecosystem health in apple-marigold intercropping system.

Despite the significant findings reported in this study, there are several limitations that need to be acknowledged. Firstly, the study was conducted over one growing season, and therefore, future studies could expand on this by examining long-term effects over multiple years. Secondly, the study was conducted in a single geographic region, so it will be important to investigate whether our findings are generalizable to other regions and soil types. Thirdly, although the study provides insights into the changes in soil metabolomics and bacterial community structures due to intercropping, the mechanisms involved in these changes require further investigation. Lastly, our experimental design only included one type of intercropping system, therefore, our findings may not be representative of other intercropping systems or plant varieties.

## Conclusion

In this study, intercropping marigold could significantly increase themoisture, enzyme activities, nutrients (OM, AN, and AP), and bacterial diversity of apple rhizospheresoils. Moreover, the marker bacteria, *Rhizobiales*, *Pseudomonadales*, and *Bacillales*, were improved in apple-marigold intercropping system. With soil metabolomics, intercropping withmarigold, carbohydrate metabolism was upregulated, and the metabolism of sucrose and starch was impacted. This work demonstrates that intercropping marigold can effectively enhance the quality of apple rhizosphere soils.

## Data availability statement

The datasets presented in this study can be found in online repositories. The names of the repository/repositories and accession number(s) can be found below: https://www.ncbi.nlm.nih.gov/, PRJNA916681.

## Author contributions

XX, RC, XZ, and XW designed the experiment. XX, RC, CX, CZ, and LD performed the experiment and analyzed the data. XX and RC wrote the manuscript. All authors contributed to the article and approved the submitted version.

## Funding

This study was financially supported by the National Natural Science Foundation of China (42007140), the Natural Science Foundation of Shandong Province (ZR2020QD121, ZR2021QC203, and ZR2021MC129); and China Agriculture Research System of MOF and MARA (CARS-27).

## Conflict of interest

The authors declare that the research was conducted in the absence of any commercial or financial relationships that could be construed as a potential conflict of interest.

## Publisher’s note

All claims expressed in this article are solely those of the authors and do not necessarily represent those of their affiliated organizations, or those of the publisher, the editors and the reviewers. Any product that may be evaluated in this article, or claim that may be made by its manufacturer, is not guaranteed or endorsed by the publisher.

## Footnotes

^1^http://huttenhower.sph.harvard.edu/galaxy
