# Supplementary material for: Apple-marigold intercropping improves soil properties by changing soil metabolomics and bacterial community structures
Source: Front Microbiol. 2023 Jun 29;14:1195985. doi: 10.3389/fmicb.2023.1195985 (PMC10343436; doi:10.3389/fmicb.2023.1195985)
Supplement: Supplementary file 1 [file Data_Sheet_1.docx]

Supplementary Material

Apple-Marigold Intercropping Improves Soil Properties by Changing Soil Metabolomics and Bacterial Community Structures

Xiaomin Xue^1,a^, Ru Chen^1,a^, Chao Xu^2^, Chunxiang Zhang^3^,Lijuan Dong^3^, Xianyan Zhao^2*^, Xiaohan Wang^2*^

^1^Shandong Institute of Pomology, Tai’an, China

^2^School of Bioengineering, Qilu University of Technology, Shandong Academy of Sciences, Jinan, China

^3^Taishan Forestry Research Institute, Tai’an, China

*** Correspondence:**

Xianyan Zhao: zhaoxianyan@qlu.edu.cn;

Xiaohan Wang: xiaohanw@qlu.edu.cn

^a^These authors contributed equally to this work.

**Supplementary Table 1**. The reads and OTUs of soil samples.

| Sample | CK1 | CK2 | CK3 | T1 | T2 | T3 |
| --- | --- | --- | --- | --- | --- | --- |
| Reads | 94607 | 143193 | 81976 | 134599 | 109706 | 152848 |
| OTUs | 79973 | 120803 | 69675 | 114506 | 93103 | 130662 |

**Supplementary Table 2**. The month details for the 2021 to 2022.

|  |  | Apr | May | Jun | Jul | Aug | Sep | Oct |
| --- | --- | --- | --- | --- | --- | --- | --- | --- |
| 2021 | Average rainfall (mm) | 2.1 | 49.7 | 215.1 | 270.5 | 241.7 | 138.9 | 43.4 |
|  | Average temperature (℃) | 13.9 | 20.6 | 26.5 | 27.5 | 25.5 | 22.5 | 14.6 |
| 2022 | Average rainfall (mm) | 91.2 | 45.6 | 135.7 | 190.1 | 344.4 | 51.4 | 12.7 |
|  | Average temperature (℃) | 16.4 | 21.0 | 27.2 | 26.9 | 27.0 | 22.3 | 13.9 |


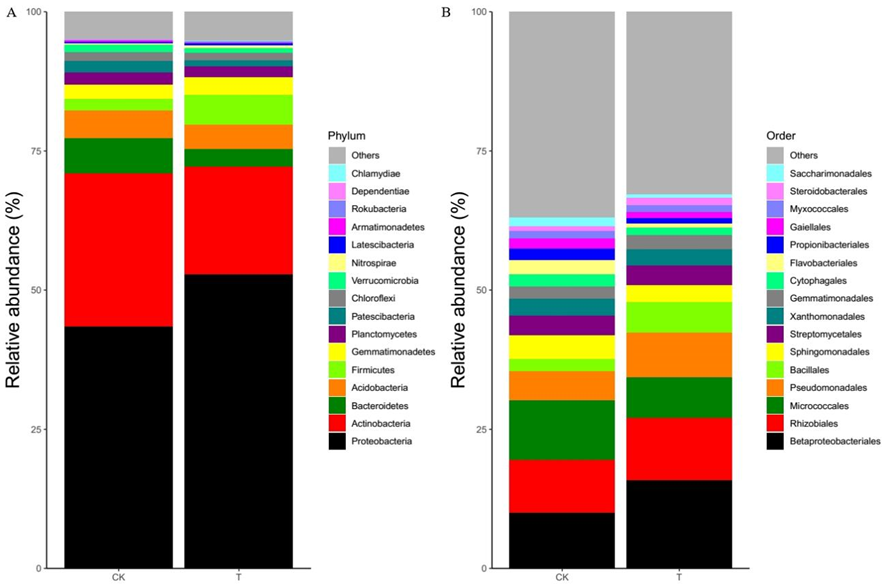


**Supplementary Figure 1.** Effects of intercropping marigold on the relative abundance of the bacterial phylum (A) and order (B) from the soil of apple orchard.


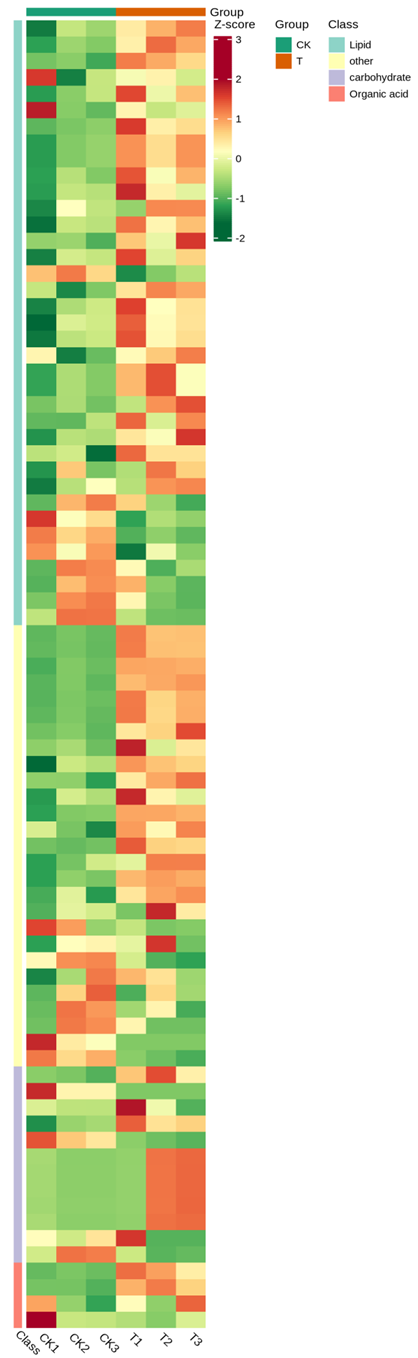


**Supplementary Figure 2.** Heat map clustering of the soil metabolites


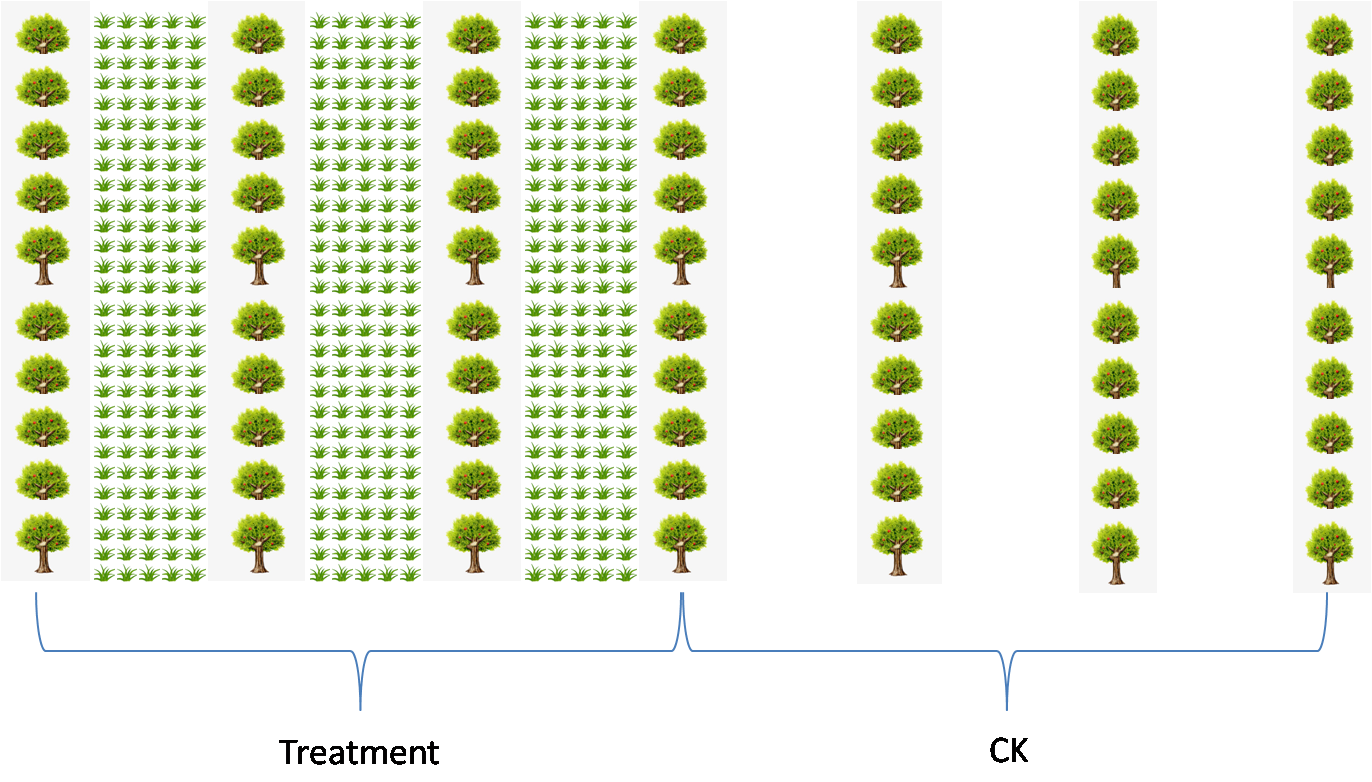


**Supplementary Figure 3.** Sketch map of intercropping and CK.
